# Cyclosporin A and verapamil have different effects on energy metabolism in multidrug-resistant tumour cells.

**DOI:** 10.1038/bjc.1990.234

**Published:** 1990-07

**Authors:** H. J. Broxterman, H. M. Pinedo, G. J. Schuurhuis, J. Lankelma

**Affiliations:** Department of Medical Oncology, Free University Hospital, Amsterdam, The Netherlands.

## Abstract

Cyclosporin A (Sandimmune) rapidly induced an increase in daunorubicin accumulation in multidrug-resistant human ovarian carcinoma cells (2780AD) and was more potent than verapamil. Steady-state 3H-cyclosporin A accumulation at 37 degrees C in 2780AD cells was 60-70% of that in the sensitive A2780 cells. A rapid increase of ATP consumption and lactate production was induced in 2780AD cells by verapamil, but not by cyclosporin A. These results suggest that the interactions of cyclosporin A and verapamil with P-glycoprotein, which leads to inhibition of drug transport, have a different mechanistic basis.


Br. J. Cancer (1990), 62, 85-88 © Macmillan Press Ltd., 1990

### Cyclosporin A and verapamil have different effects on energy metabolism in multidrug-resistant tumour cells

H.J. Broxterman, H.M. Pinedo, G.J. Schuurhuis & J. Lankelma

*Department of Medical Oncology, Free University Hospital, de Boelelaan 1117, 1081 HV Amsterdam, The Netherlands.*

**Summary** Cyclosporin A (Sandimmune®) rapidly induced an increase in daunorubicin accumulation in multidrug-resistant human ovarian carcinoma cells (2780AD) and was more potent than verapamil. Steady-state $^3$H-cyclosporin A accumulation at 37°C in 2780AD cells was 60–70% of that in the sensitive A2780 cells. A rapid increase of ATP consumption and lactate production was induced in 2780AD cells by verapamil, but not by cyclosporin A. These results suggest that the interactions of cyclosporin A and verapamil with P-glycoprotein, which leads to inhibition of drug transport, have a different mechanistic basis.

The development of resistance of tumour cells to a wide range of natural product derived anticancer agents is generally called multidrug resistance. One type of multidrug resistance is characterised by an increased energy-dependent outward cellular transport of the anti-cancer drugs (Peterson *et al.*, 1980; Skovsgaard & Nissen, 1982; Inaba & Johnson, 1978). This transport is related to overexpression of a plasmamembrane glycoprotein, termed P-glycoprotein (Bradley *et al.*, 1988), which has been shown to have ATPase activity (Hamada & Tsuruo, 1988). The discovery of this drug export system has triggered research to find drugs which specifically would block its activity, since such drugs might be potentially useful to increase the efficacy of chemotherapy. A number of classes of compounds, which increase the accumulation of cytostatic drugs, such as anthracyclines and vinca alkaloids, in P-glycoprotein expressing cell lines, have now been identified (Helson, 1984; Kessel, 1986; Schuurhuis *et al.*, 1987). Specific binding of such drugs to P-glycoprotein (Safa *et al.*, 1987), stimulation of ATPase activity of P-glycoprotein (Hamada & Tsuruo, 1988) and increase of ATP hydrolysis in multidrug resistant cells (Broxterman *et al.*, 1988*b*) by such drugs has been shown. However, the exact molecular mechanism of action of these drugs has not been elucidated (Huet & Robert, 1988; Gruber *et al.*, 1988) nor is it known whether they all act via the same mechanism (Akiyama *et al.*, 1988). Structure-activity relationships are now being determined for these drugs (Zamora *et al.*, 1988; Ramu & Ramu, 1989).

Recently it has been found that the immunosuppressant drug cyclosporin A is able to reverse P-glycoprotein-dependent multidrug resistance (Slater *et al.*, 1986; Osieka *et al.*, 1986; Twentyman *et al.*, 1987; Twentyman, 1988; Vayuvegula *et al.*, 1988), but the mechanism by which cyclosporins modulate the sensitivity to chemotherapeutic agents has not been elucidated (Chambers *et al.*, 1989). In order to delineate further the mechanism of action of cyclosporin A as a resistance modifier we compared its effect with verapamil on daunorubicin accumulation and energy metabolism in multidrug-resistant cells and found evidence that these agents represent categories of drugs which differ in their interaction with P-glycoprotein.

### Materials and methods

### Chemicals

Verapamil hydrochloride was from Sigma (St Louis, MO, USA) and daunorubicin hydrochloride was from Specia (Paris, France). Sandimmune®, which is cyclosporin A, dissolved in cremophor EL/ethanol and pure cyclosporin A (which we dissolved in ethanol) were gifts from Sandoz (Basle, Switzerland). 14-$^{14}$C-daunorubicin (spec. act. 45 Ci mol$^{-1}$) and [Mebmt-$\beta$-$^3$H]-cyclosporin A (10.5 Ci mmol$^{-1}$) were from Amersham (UK).

### Cells

Human ovarian carcinoma cells A2780 and 2780AD and culture conditions have been described (Rogan *et al.*, 1984; Broxterman *et al.*, 1987). 2780AD cells were cultured in the presence of 2 µM doxorubicin (Adriablastina, Farmitalia Carlo Erba, Milan, Italy) until 4–7 days before experiments.

### Drug accumulation experiments

Cellular steady-state accumulation of $^{14}$C-daunorubicin and $^3$H-cyclosporin A was determined by incubation of cells in complete growth medium, including 10% fetal calf serum and buffered by 20 mM Hepes, pH 7.45. After incubation cells were washed twice in ice-cold phosphate buffered saline and cell-associated radioactivity was determined with liquid scintillation counting (Broxterman *et al.*, 1987). Values are expressed as pmol per cell associated drug per $10^6$ cells after correction for 0°C direct binding to the cells.

### ATP and ADP measurements

Ribonucleoside di- and triphosphates were measured in cellular trichloroacetic acid extracts with an ion exchange high-performance liquid chromatography system (Leyva *et al.*, 1982). Cells were incubated with verapamil, cyclosporin A or vehicle in complete medium (medium A) or medium without glucose but with 10% fetal calf serum and 10 mM sodium azide (medium B) (Broxterman *et al.*, 1988*b*). Cellular lactate formation was measured as described (Broxterman *et al.*, 1989).

### Results and discussion

We first examined the potency of cyclosporin A (Sandimmune) to induce an increase in daunorubicin accumulation in 2780AD cells, because such an effect has been reported (Nooter *et al.*, 1989), while others have found no evidence for correction of daunorubicin accumulation in multidrug resistant cells (Vayuvegula *et al.*, 1988). As shown in Figure 1, Sandimmune is a more potent inducer of daunorubicin accumulation than verapamil in 2780AD cells: e.g. 2 µM Sandimmune had a similar effect as 8 µM verapamil, while 8 µM Sandimmune was more potent than 8 µM verapamil (Table I). Since we found that the vehicle Cremophor EL when used in the same concentration as present in a 2 µM Sandimmune solution (i.e. 0.003% vol./vol. or 33 µg ml$^{-1}$)

Correspondence: H.J. Broxterman.
Received 2 October 1989; and in revised form 1 March 1990.

**Figure 1** Effect of cyclosporin A (Sandimmune) on daunorubicin accumulation in 2780AD cells. Cells were incubated with 1 μM daunorubicin for 60 min at 37°C in complete medium, pH = 7.45. Results are from two experiments, mean ± s.d.

**Table I** Daunorubicin accumulation in 2780AD cells

| *Drug* | *(μM)* | *Accumulation (pmol per $10^6$ cells)* |
|---|---|---|
| – | | 19 ± 3 |
| + verapamil | (8) | 113 ± 2 |
| + Sandimmune | (8) | 241 ± 9 |
| + cyclosporin A | (8) | 325(*n* = 1) |

About 200,000 cells were incubated (60 min, 37°C) in 550 μl medium A with 1 μM $^{14}$C-daunorubicin. Data are means ± s.d. of three experiments, each performed in quadruplicate.

itself caused a small, but significant increase of daunorubicin accumulation in 2780AD cells, we have checked in a separate experiment that 8 μM cyclosporin A (dissolved in ethanol) had a similar effect as 8 μM Sandimmune (Table I). The effects of Cremophor EL on doxorubicin and vincristine cytotoxicity in multidrug resistant cells have been further analysed (Schuurhuis *et al.*, submitted).

Since 2780AD is a typical multidrug resistant cell line, in which the defect of daunorubicin transport is related to overexpression of the *mdr1* gene (Van der Bliek *et al.*, 1988; Broxterman *et al.*, 1988*a*), cyclosporin A apparently is a potent modulator of P-glycoprotein mediated drug transport. A number of agents that reverse multidrug resistance have been suggested to interact competitively with antitumour agents such as the vinca alkaloids for the P-glycoprotein associated transport mechanism (Horio *et al.*, 1988). Therefore, to find evidence for P-glycoprotein dependent transport of cyclosporin A itself, we measured the accumulation of cyclosporin A in A2780 and 2780AD cells and found (Table II) that the accumulation of $^3$H-cyclosporin A in 2780AD cells was 60–70% of that in A2780 cells. Although, the solubiliser Cremophor EL tended to cause a higher cyclosporin A accumulation in both cell types, a difference in accumulation between parent and resistant cells was present, whether Cremophor EL was present in the medium (in Sandimmune) or not, although in the latter case no statistical significance was reached (Table II). We found similar accumulation differences for AUXB1 and CH$^r$C5 cells, another couple of sensitive and multidrug resistant cells (not shown). These data, together with the recent finding that cyclosporin A binds specifically to P-glycoprotein (Safa *et al.*, 1989; Foxwell *et al.*, 1989) would suggest that cyclosporin A itself is a substrate for the P-glycoprotein related transport mechanism.

**Table II** Cyclosporin A accumulation in 2780 cells

| | *Accumulation (pmol per $10^6$ cells)* | |
|---|---|---|
| *Drug* | *A2780* | *2780AD* |
| Sandimmune | 88 ± 17 | 56 ± 7$^a$ |
| Cyclosporin A | 46 ± 8 | 33 ± 6$^b$ |

About 1,000,000 cells were incubated in medium A at 37°C with $^3$H-cylcosporin A, diluted with Sandimmune® or cyclosporin A (stock in ethanol) to 2 μM cyclosporin A final concentration. From time-curves it appeared that a steady-state was reached in 2 min incubation. Data are from 60 min incubations, means ± s.d. from 5 (Sandimmune) or 2 (cyclosporin A) separate experiments. $^aP < 0.01$; $^bP > 0.05$, compared to A2780 (Student's *t* test). In three experiments the effect of 16 μM verapamil on cyclosporin A accumulation in 2780AD cells was measured; a small increase (122 ± 13% of controls) was measured.

We did find a small effect of 16 μM verapamil on the accumulation of cyclosporin A in 2780AD cells (legend Table II), while it had a large effect on daunorubicin accumulation in 2780AD cells (Figure 1). Goldberg *et al.* (1988) found a more pronounced effect of verapamil on cyclosporin A accumulation in CH$^R$C5 cells at a higher verapamil/cyclosporin concentration ratio than we used. Therefore some interaction between these drugs at P-glycoprotein level may still be present.

To delineate further the mechanism of reversal of resistance by cyclosporin A we investigated the effect of the drug on energy metabolism in 2780 cells. We have previously shown that resistance modifiers such as verapamil, bepridil, trifluoperazine and diltiazem under appropriate conditions can decrease the ATP/ADP ratio in P-glycoprotein overexpressing cells (Broxterman *et al.*, 1988*b*), and in addition we showed that the same compounds induced a specific increase in lactate formation rate in multidrug-resistant, but not in sensitive cells (Broxterman *et al.*, 1989). Together with data showing that these drugs are potent inhibitors of ATP-dependent vinblastine binding and transport in P-glycoprotein containing plasmamembrane vesicles (Horio *et al.*, 1988), these results suggested that resistance modifying agents themselves may be transported by the P-glycoprotein dependent pump system. Table III, however, shows that 4 μM (and 8 μM, not shown) Sandimmune, which more effectively reversed daunorubicin accumulation than 8 μM verapamil (Figure 1), did not affect the ATP/ADP ratio in 2780AD cells, while 8 μM verapamil decreased this ratio to 48%. In a separate experiment we have checked that the onset of cyclosporin's effect on daunorubicin accumulation was very rapid, that is within 2.5 min. Furthermore a steady-state accumulation of cyclosporin A was already reached in 2 min (legend of Table II). Thus if a transport-related energy effect of cyclosporin A would be present, it should have a rapid onset too. Furthermore we found that cyclosporin A in contrast to verapamil (Broxterman *et al.*, 1989), did not induce an increase in glycolysis during 60 min of drug exposure in three multidrug resistant cell lines, 2780AD (Table IV), H134AD and MCF-7/Adr$^R$ (not shown). A small effect of cremophor EL on lactate formation was seen in A2780 only.

In conclusion, we present evidence that cyclosporin A is a very effective inhibitor of the P-glycoprotein related dauno-

**Table III** Effect of cyclosporin A and verapamil on ATP/ADP ratio in 2780 cells

| | | *ATP:ADP* | |
|---|---|---|---|
| *Drug* | *Concentration (μM)* | *A2780* | *2780AD* |
| Verapamil | 8 | 98 ± 11%$^a$ | 48 ± 10% |
| Sandimmune | 4 | 98 ± 16% | 97 ± 6% |

Cells were incubated in medium with sodium azide (medium B, as described in Broxterman *et al.*, 1988*b*) during 7.5 min. $^a$Data are expressed as percentage of control samples (no drug added; mean ± s.d. of 2 experiments) incubated for 7.5 min in medium B. In these control cells ATP levels decreased to about 40% compared to cells incubated in complete growth medium.

**Table IV** Effect of cyclosporin A and verapamil on lactate formation in 2780 cells

| | | *Lactate % of control* | |
|---|---|---|---|
| *Drug* | *(μM)* | *A2780* | *2780AD* |
| Verapamil | 8 | 101± 2 | 135±3 |
| Sandimmune | 8 | 113± 7 | 99±5 |
| Cyclosporin A | 8 | 106±10 | 102±5 |
| Cremophor EL | 132 μg/ml[a] | 117±11 | 101±2 |

Cell suspensions of about $2 \times 10^6$ cells $ml^{-1}$ were incubated in medium with 10% dialysed fetal calf serum (pH = 7.45) for 60 min at 37°C. Data are means±s.d. from three separate experiments, each performed in triplicate. [a]Concentration present in 8 μM Sandimmune.

rubicin transport across the plasmamembrane, but apparently does not increase the cellular energy demand for its interaction with P-glycoprotein. Evidence for a direct interaction of cyclosporin A with P-glycoprotein comes from the observation that this drug competitively inhibits the binding of a photoaffinity-labelled vinblastine analogue to P-glycoprotein and of ATP-dependent vincristine binding to plasma membranes of multidrug resistant cells with an apparent inhibition constant of 0.1 μM (Safa *et al.*, 1989). The present study suggests that there are differences between the mechanism of interaction of cyclosporin A and verapamil with P-glycoprotein.

Cyclosporin A is a hydrophobic peptide and has been shown to partition into phospholipid vesicles and to increase membrane fluidity (Haynes *et al.*, 1985). We have also found, using 1,6-diphenyl-1,3,5-hexatriene fluorescence polarisation, that cyclosporin increased plasmamembrane fluidity of A2780 and 2780AD cells (unpublished observations). Thus by disrupting membrane architecture cyclosporin might affect P-glycoprotein function (Arsenault *et al.*, 1988). Alternatively, inhibition of protein kinase C activity by cyclosporin A (Walker *et al.*, 1989) might interfere differently with the energizing of P-glycoprotein.

This study was supported by a Bristol-Myers research grant and the Dutch Cancer Society (IKA-VU 88-22).

### References

AKIYAMA, S.-I., CORNWALL, M.M., KUWANO, M., PASTAN, I. & GOTTESMAN, M.M. (1988). Most drugs that reverse multidrug resistance also inhibit photoaffinity labeling of P-glycoprotein by a vinblastine analog. *Mol. Pharmacol.*, **33**, 144.

ARSENAULT, A.L., LING, V. & KARTNER, N. (1988). Altered plasma membrane ultrastructure in multidrug-resistant cells. *Biochim. Biophys. Acta*, **938**, 315.

BRADLEY, G., JURANKA, P.F. & LING, V. (1988). Mechanism of multidrug resistance. *Biochim. Biophys. Acta*, **948**, 87.

BROXTERMAN, H.J., KUIPER, C.M., SCHUURHUIS, G.J., TSURUO, T., PINEDO, H.M. & LANKELMA, J. (1988*a*). Increase of daunorubicin and vincristine accumulation in multidrug resistance human ovarian carcinoma cells by a monoclonal antibody reacting with P-glycoprotein. *Biochem. Pharmacol.*, **37**, 2389.

BROXTERMAN, H.J., KUIPER, C.M., SCHUURHUIS, G.J. VAN DER HOEVEN, J.J.M., PINEDO, H.M. & LANKELMA, J. (1987). Daunomycin accumulation in resistant tumor cells as a screening model for resistance modifying drugs: role of protein binding. *Cancer Lett.*, **35**, 87.

BROXTERMAN, H.J., PINEDO, H.M., KUIPER, C.M., KAPTEIN, L.C.M., SCHUURHUIS, G.J. & LANKELMA, J. (1988*b*). Induction by verapamil of a rapid increase in ATP consumption in multidrug-resistant tumor cells. *FASEB J.*, **2**, 2278.

BROXTERMAN, H.J., PINEDO, H.M., KUIPER, C.M., SCHUURHUIS, G.J. & LANKELMA, J. (1989). Glycolysis in P-glycoprotein-overexpressing human tumor cell lines. Effects of resistance-modifying agents. *FEBS Lett.*, **247**, 405.

CHAMBERS, S.K., HAIT, W.N., KACINSKI, B.M., KEYES, S.R. & HANDSCHUMACHER, R.E. (1989). Enhancement of anthracycline growth inhibition in parent and multidrug-resistant Chinese hamster ovary cells by cyclosporin A and its analogues. *Cancer Res.*, **49**, 6275.

FOXWELL, B.M.J., MACKIE, A., LING, V. & RYFFEL, B. (1989). Identification of the multidrug resistance-related P-glycoprotein as a cyclosporin binding protein. *Mol. Pharmacol.*, **36**, 543.

GOLDBERG, H., LING, V., WONG, P.Y. & SKORECKI, K. (1988). Reduced cyclosporin accumulation in multidrug-resistant cells. *Biochem. Biophys. Res. Commun.*, **152**, 552.

GRUBER, A., PETERSON, C. & REIZENSTEIN, P. (1988). D-verapamil and L-verapamil are equally effective in increasing vincristine accumulation in leukemic cells *in vitro*. *Int. J. Cancer*, **41**, 224.

HAMADA, H. & TSURUO, T. (1988). Characterization of the ATPase activity of the Mr 170,000 to 180,000 membrane glycoprotein (P-glycoprotein) associated with multidrug resistance in K562/ADM cells. *Cancer Res.*, **48**, 4926.

HAYNES, M., FULLER, L., HAYNES, D.H. & MILLER, J. (1985). Cyclosporin partitions into phospholipid vesicles and disrupts membrane architecture. *Immunol. Lett.*, **11**, 343.

HELSON, L. (1984). Calcium channel-blocker enhancement of anticancer drug cytotoxicity – a review. *Cancer Drug Deliv.*, **1**, 353.

HORIO, M., GOTTESMAN, M.M. & PASTAN, I. (1988). ATP-dependent transport of vinblastine in vesicles from human multidrug-resistant cells. *Proc. Natl Acad. Sci. USA*, **85**, 3580.

HUET, S. & ROBERT, J. (1988). The reversal of doxorubicin resistance by verapamil is not due to an effect on calcium channels. *Int. J. Cancer*, **41**, 283.

INABA, M. & JOHNSON, R.K. (1978). Uptake and retention of adriamycin and daunorubicin by sensitive and anthracycline-resistant sublines of P388 leukemia. *Biochem. Pharmacol.*, **27**, 2123.

KESSEL, D. (1986). Circumvention of resistance to anthracyclines by calcium antagonists and other membrane-perturbing agents. *Cancer Surveys*, **5**, 109.

LEYVA, A., APPEL, H. & PINEDO, H.M. (1982). Purine modulation of thymidine activity in L1210 leukemia cells *in vitro*. *Leukemia Res.*, **6**, 483.

NOOTER, K., OOSTRUM, R., JONKER, R., VAN DEKKEN, H., STOKDIJK, W. & VAN DEN ENGH, G. (1989). Effect of cyclosporin A on daunorubicin accumulation in multidrug-resistant P388 leukemia cells measured by real-time flow cytometry. *Cancer Chemother. Pharmacol.*, **23**, 296.

OSIEKA, R., SEEBER, S., PANNENBACKER, R., SOLL, D., GLATTE, P. & SCHMIDT, C.G. (1986). Enhancement of etoposide-induced cytotoxicity by cyclosporin A. *Cancer Chemother. Pharmacol.*, **18**, 198.

PETERSON, C., BAURAIN, R. & TROUET, A. (1980). The mechanism for cellular uptake, storage and release of daunorubicin. Studies on fibroblasts in culture. *Biochem. Pharmacol.*, **29**, 1687.

RAMU, N. & RAMU, A. (1989). Circumvention of adriamycin resistance by dipyridamole analogues: a structure-activity relationship study. *Int. J. Cancer*, **43**, 487.

ROGAN, A.M., HAMILTON, T.C., YOUNG, R.C., KLECKER, R.W. & OZOLS, R.F. (1984). Reversal of adriamycin resistance by verapamil in human ovarian cancer. *Science*, **224**, 994.

SAFA, A.R., CHOE, M.M., MORROW, M. & MANLEY, S.A. (1989). Cyclosporin A and its non-immunosuppressive analogs reverse Vinca alkaloid resistance by interacting with P-glycoprotein. *Proc. Am. Assoc. Cancer Res.*, **30**, 498.

SAFA, A.R., GLOVER, C.J., SEWELL, J.L., MEYERS, M.B., BIEDLER, J.L. & FELSTED, R.L. (1987). Identification of the multidrug resistance-related membrane glycoprotein as an acceptor for calcium channel blockers. *J. Biol. Chem.*, **262**, 7884.

SCHUURHUIS, G.J., BROXTERMAN, H.J., VAN DER HOEVEN, J.J.M., PINEDO, H.M. & LANKELMA, J. (1987). Potentiation of doxorubicin cytotoxicity by the calcium antagonist bepridil in anthracycline-resistant and sensitive cell lines. *Cancer Chemother. Pharmacol.*, **20**, 285.

SKOVSGAARD, T. & NISSEN, N.I. (1982). Membrane transport of anthracyclines. *Pharmac. Ther.*, **18**, 293.

SLATER, L.M., SWEET, P., STUPECKI, M., WETZEL, M.W. & GUPTA, S. (1986). Cyclosporin A corrects daunorubicin resistance in Ehrlich ascites carcinoma. *Br. J. Cancer*, **54**, 235.

TWENTYMAN, P.R. (1988). A possible role for cyclosporins in cancer chemotherapy. *Anticancer Res.*, **8**, 985.

TWENTYMAN, P.R., FOX, N.E. & WHITE, D.J.G. (1987). Cyclosporin A and its analogues as modifiers of adriamycin and vincristine resistance in a multidrug resistant human lung cancer cell line. *Br. J. Cancer*, **56**, 55.

VAN DER BLIEK, A.M., BAAS, F., VAN DER VELDE-KOERTS, T. & 6 others (1988). Genes amplified and overexpressed in human multidrug resistant cell lines. *Cancer Res.*, **48**, 5927.

VAYUVEGULA, B., SLATER, L., MEADOR, J. & GUPTA, S. (1988). Correction of altered plasma membrane potentials. A possible mechanism of cyclosporin A and verapamil reversal of pleiotropic drug resistance in neoplasia. *Cancer Chemother. Pharmacol.*, **22**, 163.

WALKER, R.J., LAZZARO, V.A., DUGGIN, G.G., HORVATH, J.S. & TILLER, D.J. (1989). Cyclosporin A inhibits protein kinase C activity: a contributing mechanism in the development of nephrotoxicity? *Biochem. Biophys. Res. Commun.*, **160**, 409.

ZAMORA, J.M., PEARCE, H.L., & BECK, W.T. (1988). Physical-chemical properties shared by compounds that modulate multidrug resistance in human leukemic cells. *Mol. Pharmacol.*, **33**, 454.


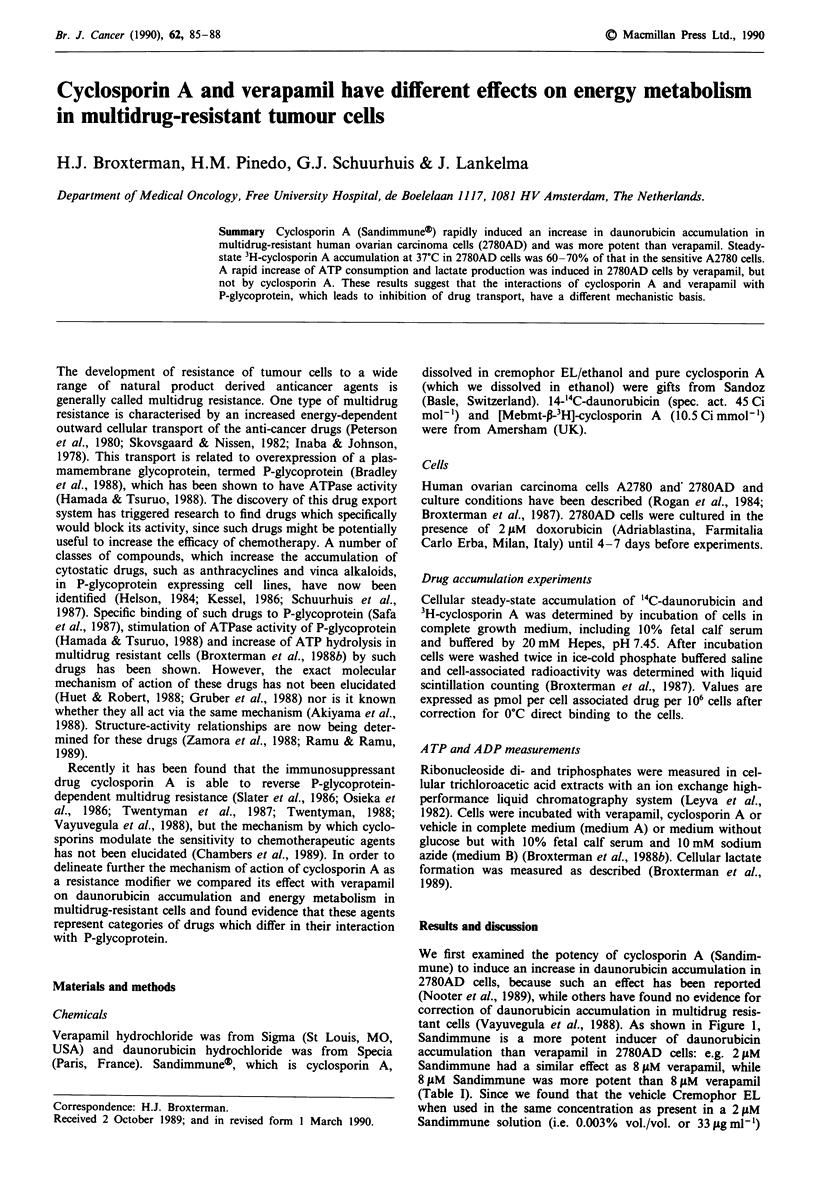

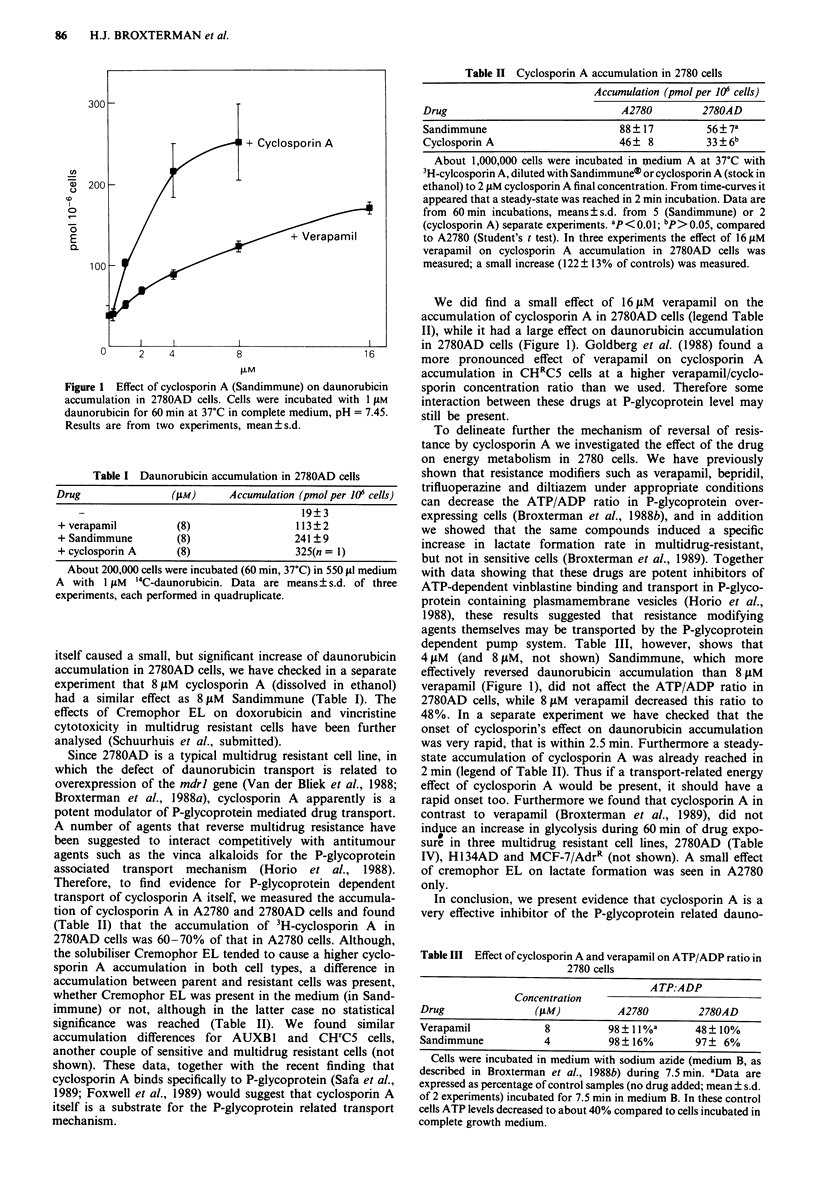

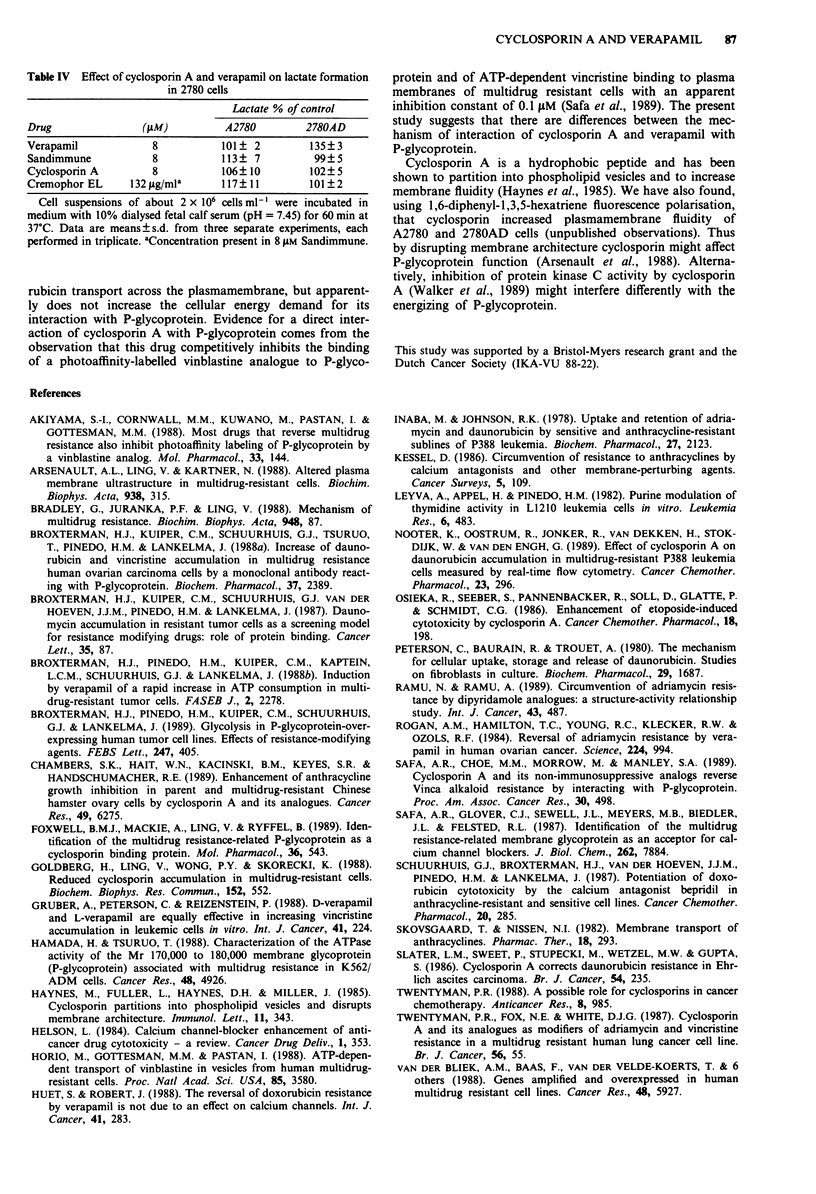

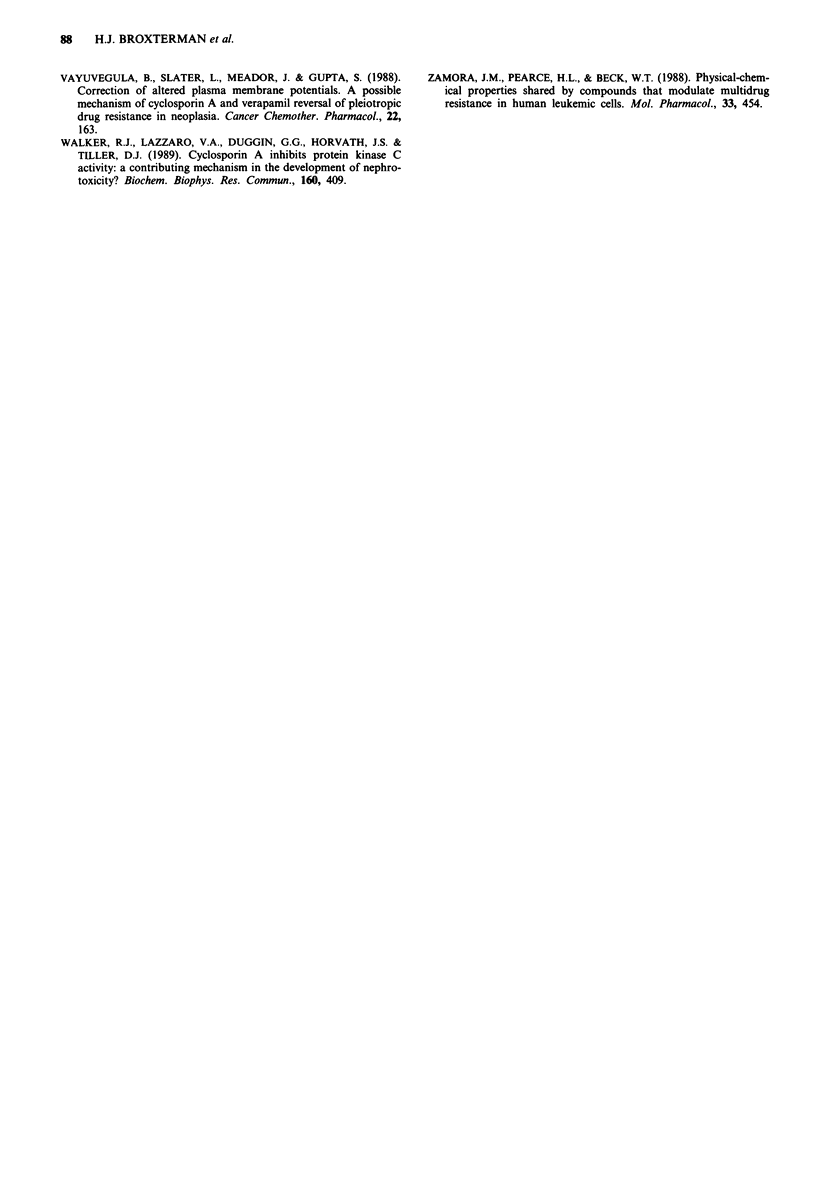


## References

[OCR_00335] Akiyama S., Cornwell M. M., Kuwano M., Pastan I., Gottesman M. M. (1988). Most drugs that reverse multidrug resistance also inhibit photoaffinity labeling of P-glycoprotein by a vinblastine analog.. Mol Pharmacol.

[OCR_00341] Arsenault A. L., Ling V., Kartner N. (1988). Altered plasma membrane ultrastructure in multidrug-resistant cells.. Biochim Biophys Acta.

[OCR_00346] Bradley G., Juranka P. F., Ling V. (1988). Mechanism of multidrug resistance.. Biochim Biophys Acta.

[OCR_00350] Broxterman H. J., Kuiper C. M., Schuurhuis G. J., Tsuruo T., Pinedo H. M., Lankelma J. (1988). Increase of daunorubicin and vincristine accumulation in multidrug resistant human ovarian carcinoma cells by a monoclonal antibody reacting with P-glycoprotein.. Biochem Pharmacol.

[OCR_00359] Broxterman H. J., Kuiper C. M., Schuurhuis G. J., van der Hoeven J. J., Pinedo H. M., Lankelma J. (1987). Daunomycin accumulation in resistant tumor cells as a screening model for resistance modifying drugs: role of protein binding.. Cancer Lett.

[OCR_00364] Broxterman H. J., Pinedo H. M., Kuiper C. M., Kaptein L. C., Schuurhuis G. J., Lankelma J. (1988). Induction by verapamil of a rapid increase in ATP consumption in multidrug-resistant tumor cells.. FASEB J.

[OCR_00370] Broxterman H. J., Pinedo H. M., Kuiper C. M., Schuurhuis G. J., Lankelma J. (1989). Glycolysis in P-glycoprotein-overexpressing human tumor cell lines. Effects of resistance-modifying agents.. FEBS Lett.

[OCR_00376] Chambers S. K., Hait W. N., Kacinski B. M., Keyes S. R., Handschumacher R. E. (1989). Enhancement of anthracycline growth inhibition in parent and multidrug-resistant Chinese hamster ovary cells by cyclosporin A and its analogues.. Cancer Res.

[OCR_00383] Foxwell B. M., Mackie A., Ling V., Ryffel B. (1989). Identification of the multidrug resistance-related P-glycoprotein as a cyclosporine binding protein.. Mol Pharmacol.

[OCR_00388] Goldberg H., Ling V., Wong P. Y., Skorecki K. (1988). Reduced cyclosporin accumulation in multidrug-resistant cells.. Biochem Biophys Res Commun.

[OCR_00393] Gruber A., Peterson C., Reizenstein P. (1988). D-verapamil and L-verapamil are equally effective in increasing vincristine accumulation in leukemic cells in vitro.. Int J Cancer.

[OCR_00397] Hamada H., Tsuruo T. (1988). Characterization of the ATPase activity of the Mr 170,000 to 180,000 membrane glycoprotein (P-glycoprotein) associated with multidrug resistance in K562/ADM cells.. Cancer Res.

[OCR_00403] Haynes M., Fuller L., Haynes D. H., Miller J. (1985). Cyclosporin partitions into phospholipid vesicles and disrupts membrane architecture.. Immunol Lett.

[OCR_00408] Helson L. (1984). Calcium channel blocker enhancement of anticancer drug cytotoxicity--a review.. Cancer Drug Deliv.

[OCR_00411] Horio M., Gottesman M. M., Pastan I. (1988). ATP-dependent transport of vinblastine in vesicles from human multidrug-resistant cells.. Proc Natl Acad Sci U S A.

[OCR_00416] Huet S., Robert J. (1988). The reversal of doxorubicin resistance by verapamil is not due to an effect on calcium channels.. Int J Cancer.

[OCR_00421] Inaba M., Johnson R. K. (1978). Uptake and retention of adriamycin and daunorubicin by sensitive and anthracycline-resistant sublines of P388 leukemia.. Biochem Pharmacol.

[OCR_00426] Kessel D. (1986). Circumvention of resistance to anthracyclines by calcium antagonists and other membrane-perturbing agents.. Cancer Surv.

[OCR_00431] Leyva A., Appel H., Pinedo H. M. (1982). Purine modulation of thymidine activity in L1210 leukemia cells in vitro.. Leuk Res.

[OCR_00438] Nooter K., Oostrum R., Jonker R., van Dekken H., Stokdijk W., van den Engh G. (1989). Effect of cyclosporin A on daunorubicin accumulation in multidrug-resistant P388 leukemia cells measured by real-time flow cytometry.. Cancer Chemother Pharmacol.

[OCR_00443] Osieka R., Seeber S., Pannenbäcker R., Soll D., Glatte P., Schmidt C. G. (1986). Enhancement of etoposide-induced cytotoxicity by cyclosporin A.. Cancer Chemother Pharmacol.

[OCR_00449] Peterson C., Baurain R., Trouet A. (1980). The mechanism for cellular uptake, storage and release of daunorubicin. Studies on fibroblasts in culture.. Biochem Pharmacol.

[OCR_00454] Ramu N., Ramu A. (1989). Circumvention of adriamycin resistance by dipyridamole analogues: a structure-activity relationship study.. Int J Cancer.

[OCR_00459] Rogan A. M., Hamilton T. C., Young R. C., Klecker R. W., Ozols R. F. (1984). Reversal of adriamycin resistance by verapamil in human ovarian cancer.. Science.

[OCR_00470] Safa A. R., Glover C. J., Sewell J. L., Meyers M. B., Biedler J. L., Felsted R. L. (1987). Identification of the multidrug resistance-related membrane glycoprotein as an acceptor for calcium channel blockers.. J Biol Chem.

[OCR_00476] Schuurhuis G. J., Broxterman H. J., van der Hoeven J. J., Pinedo H. M., Lankelma J. (1987). Potentiation of doxorubicin cytotoxicity by the calcium antagonist bepridil in anthracycline-resistant and -sensitive cell lines. A comparison with verapamil.. Cancer Chemother Pharmacol.

[OCR_00483] Skovsgaard T., Nissen N. I. (1982). Membrane transport of anthracyclines.. Pharmacol Ther.

[OCR_00487] Slater L. M., Sweet P., Stupecky M., Wetzel M. W., Gupta S. (1986). Cyclosporin A corrects daunorubicin resistance in Ehrlich ascites carcinoma.. Br J Cancer.

[OCR_00492] Twentyman P. R. (1988). A possible role for cyclosporins in cancer chemotherapy.. Anticancer Res.

[OCR_00496] Twentyman P. R., Fox N. E., White D. J. (1987). Cyclosporin A and its analogues as modifiers of adriamycin and vincristine resistance in a multi-drug resistant human lung cancer cell line.. Br J Cancer.

[OCR_00502] Van der Bliek A. M., Baas F., Van der Velde-Koerts T., Biedler J. L., Meyers M. B., Ozols R. F., Hamilton T. C., Joenje H., Borst P. (1988). Genes amplified and overexpressed in human multidrug-resistant cell lines.. Cancer Res.

[OCR_00509] Vayuvegula B., Slater L., Meador J., Gupta S. (1988). Correction of altered plasma membrane potentials. A possible mechanism of cyclosporin A and verapamil reversal of pleiotropic drug resistance in neoplasia.. Cancer Chemother Pharmacol.

[OCR_00516] Walker R. J., Lazzaro V. A., Duggin G. G., Horvath J. S., Tiller D. J. (1989). Cyclosporin A inhibits protein kinase C activity: a contributing mechanism in the development of nephrotoxicity?. Biochem Biophys Res Commun.

[OCR_00522] Zamora J. M., Pearce H. L., Beck W. T. (1988). Physical-chemical properties shared by compounds that modulate multidrug resistance in human leukemic cells.. Mol Pharmacol.

